# A multicentric observational study of imaging findings in COVID-19-related rhino-orbito-cerebral mucormycosis: a new Pandora’s box

**DOI:** 10.1186/s43055-021-00631-w

**Published:** 2021-10-20

**Authors:** Annu Singhal, Shikha Jain, Swati Sharma, Vivek Cherumanalil Kottiyath, Girish Khandelwal

**Affiliations:** 1 Department of Radiodiagnosis, Atal Bihari Vajpayee Institute of Medical Sciences and Dr. Ram Manohar Lohia Hospital, New Delhi, Delhi India; 2 Department of Ophthalmology, Atal Bihari Vajpayee Institute of Medical Sciences and Dr. Ram Manohar Lohia Hospital, New Delhi, Delhi India; 3 Sky Diagnostics, New Delhi, Delhi India; 4 Aanand Diagnostics, Kota, Rajasthan India

**Keywords:** Rhino-orbito-cerebral mucormycosis, COVID-19 infection, Periantral extension, Sinonasal imaging

## Abstract

**Background:**

There is a sudden rise of fungal infection with corona virus disease. This is attributed to the immunomodulation by the disease and the drugs used, diabetes mellitus, steroid use, oxygen inhalation using dirty water, use of zinc and iron supplements, etc. Early diagnosis and prompt medical and surgical intervention is the mainstay of treatment. This can greatly reduce the high morbidity and mortality associated with this disease. The objective of the study is to describe the imaging findings of acute invasive rhino-orbito-cerebral mucormycosis (ROCM) in 25 patients with severe acute respiratory syndrome corona virus 2, from three different centers with proven mucormycosis. Special emphasis is placed on the signal enhancement patterns of sinonasal mucosa, the earliest and most common findings. Statistical analysis was performed using descriptive statistics.

**Results:**

Computed tomography (CT) and magnetic resonance imaging (MRI) of 25 patients showed most commonly involved sinuses as maxillary and ethmoid sinuses (19, 76%) together. Sino-nasal mucosal thickening was the most common finding (24, 96%). Periantral infiltration (18, 72%) preceded before orbital (15, 60%), cerebral (5, 20%) and vascular (2, 8%) complications, with grossly intact bones. Sinus wall erosions were seen in only 2 patients (8%). Palatal (22%) and maxillary alveolar arch erosion (39%) were frequent findings. CT showed minimally enhancing hypodense soft tissue thickening as the predominant finding in involved areas, while MRI showed T1 and T2 iso- to hypointense mucosal thickening (62%) and intense (43%) and no (33%) contrast enhancement as the main finding.

**Conclusions:**

Contrast enhanced MRI is better at demonstrating early mucosal abnormalities, turbinate necrosis, non-enhancing devitalized tissues, orbital apex involvement and intra-cerebral extension. Imaging findings of inflammatory tissue infiltration adjacent to the paranasal sinuses in premaxillary, retroantral fat, facial muscles, pterygopalatine fossa, temporal, infratemporal fossa and extraconal orbital fat along with typical patterns of sinonasal mucosal enhancement should raise the suspicion of acute invasive fungal etiology given the short duration of history and immunocompromised status. High incidence of periantral and orbital extension of the disease is suggestive of acute invasive form of fungal infection. Also the rapidly progressive inflammatory changes without much bone involvement should suggest the suspicion of ROCM. Bony, cerebral and vascular involvements are relatively late complications.

**Supplementary Information:**

The online version contains supplementary material available at 10.1186/s43055-021-00631-w.

## Background

Corona virus disease-19 pandemic is an outbreak of coronavirus infection that has spread rapidly on a global scale since 2019 [1]. It has significant morbidity and mortality. However, no definitive treatment has been proposed yet. Secondary bacterial and fungal infections pose further challenges in moderately and severely ill patients. Fungal infections are more common in those patients who are treated with corticosteroids, immunosuppressive drugs or supplementary oxygen and in diabetics [2]. As the nature of the disease itself is still not understood completely, it remains unanswered that whether the fungal infections are the outcome of complications of the disease or its management [3]. Invasive pulmonary Aspergillosis complicating the course of COVID-19 is widely recognized [4]. However, there has been a tremendous increase in number of cases of rhino-orbito-cerebral involvement with mucor in the COVID era, as reported from India. It is well established that management of ROCM involves early clinical and radiological diagnosis, reversal of underlying risk factors, prompt antifungal therapy and surgical debridement when indicated [5]. Ours is a descriptive multicentric observational study. There is a paucity of data in the literature on the imaging findings in ROCM in COVID-19 patients as most of it is in the form of case reports or reviews which are retrospective in nature [1–5].

## Methods

We report the sinonasal, orbital and neuroimaging findings in 25 patients of suspected acute invasive ROCM. A total of 28 scans were analyzed Three were repeat scans done after conservative management. The study comprises of cases performed at two different imaging centers and a tertiary care hospital from April 25, 2021 to May 25, 2021. All the patients had positive reverse transcriptase polymerase chain reaction test for severe acute respiratory syndrome corona virus 2 and were hospitalized with clinically severe disease as per the guidelines laid down during the second wave in India. They were on intravenous steroids and oxygen. Thirteen patients (52%) had diabetes mellitus. All of them presented with headache, facial and/or orbital pain with decreased vision, during the course of treatment. CT or MRI examination of the paranasal sinuses, orbits and brain was done, with intravenous contrast wherever possible. In four patients, contrast could not be given due to deranged renal function. The presence of mucormycosis was confirmed by histological diagnosis in all of them following clinico-radiological diagnosis of acute invasive ROCM.

### Imaging

CT scans were performed on multislice Siemens or GE machines using a routine paranasal sinuses protocol with 120 kV and 150–180 mA tube current. Intravenous contrast (low osmolar, non-ionic, 300 mg/ml Iodine content) was used routinely.

Multiplanar MR imaging was performed on 1.5 T or 3.0 T Siemens or GE MRI machines for brain, orbit and paranasal sinuses. T1 weighted, T2 weighted, fluid-attenuated inversion recovery (FLAIR) and post-contrast T1 weighted images were obtained. Diffusion weighted images were also obtained.

### Image interpretation

All the cases were assessed for involvement of the paranasal sinuses, nasal cavities, orbits and brain. On CT, partial/ complete sinus opacification, inflammatory changes, enhancement patterns and bony erosions were evaluated. On MRI, signal alterations in the mucosa and patterns of enhancement after intravenous contrast agent were evaluated. Involvement of the periantral soft tissues, orbits, brain parenchyma and adjacent bones was also assessed. The presence of any vascular complication was also noted and described. The imaging findings were broadly categorized into five groups based on the extent of regional involvement, namely sino-nasal, periantral, orbital, bony and intracranial and vascular involvement. Descriptive statistical methods were used for analysis. Image analysis was done by two radiologists and interobserver agreement was calculated using Cohen’s Kappa.

## Results

### Demographic results

A total of 25 examinations were included in the study. There were 15 males (60%) and 10 females (40%) with ages ranging from 17 to 78 years. The majority of patients (60%) were between 40–60 years of age group. Fourteen patients (56%) underwent MRI and 11 patients (44%) underwent CT scan. All the MRI examinations used intravenous contrast. Four of the CT examinations (36%) were done without contrast due to impaired renal function in these patients.

In majority of the patients, more than one of ipsilateral sinuses was involved (Table 1). The most commonly involved sinuses were maxillary and ethmoid sinuses together in 19 patients (76%), followed by maxillary, ethmoid and sphenoid in combination in 18 patients (72%). Sino-nasal mucosal thickening was the most common finding in almost all the cases (96%). Thirteen patients (54%) showed variable sinus contents. Air–fluid level was seen in 5 patients (20%), all in the maxillary sinuses. Periantral infiltration was seen in 18 patients (72%), whereas orbital extension of inflammation was seen in 15 (60%). Cerebral and vascular complications were noted only in 5 (20%) and 2 patients (8%), respectively.

**Table 1:** Regional involvement (*n* = 25)

Region involved	*n* = 25	Percentage (%)
Maxillary	24	96
Ethmoid	20	80
Sphenoid	18	72
Frontal	12	48
Nasal cavity	8	32
Nasal septum	0	0
Turbinate	5	20
Maxillary + Ethmoid	19	76
Maxillary + Ethmoid + Sphenoid	18	72
Maxillary + Ethmoid + Sphenoid + Frontal	10	40
Maxillary + Ethmoid + Sphenoid + Frontal + Nasal cavity	6	24

### Imaging

**Fig. 1:**
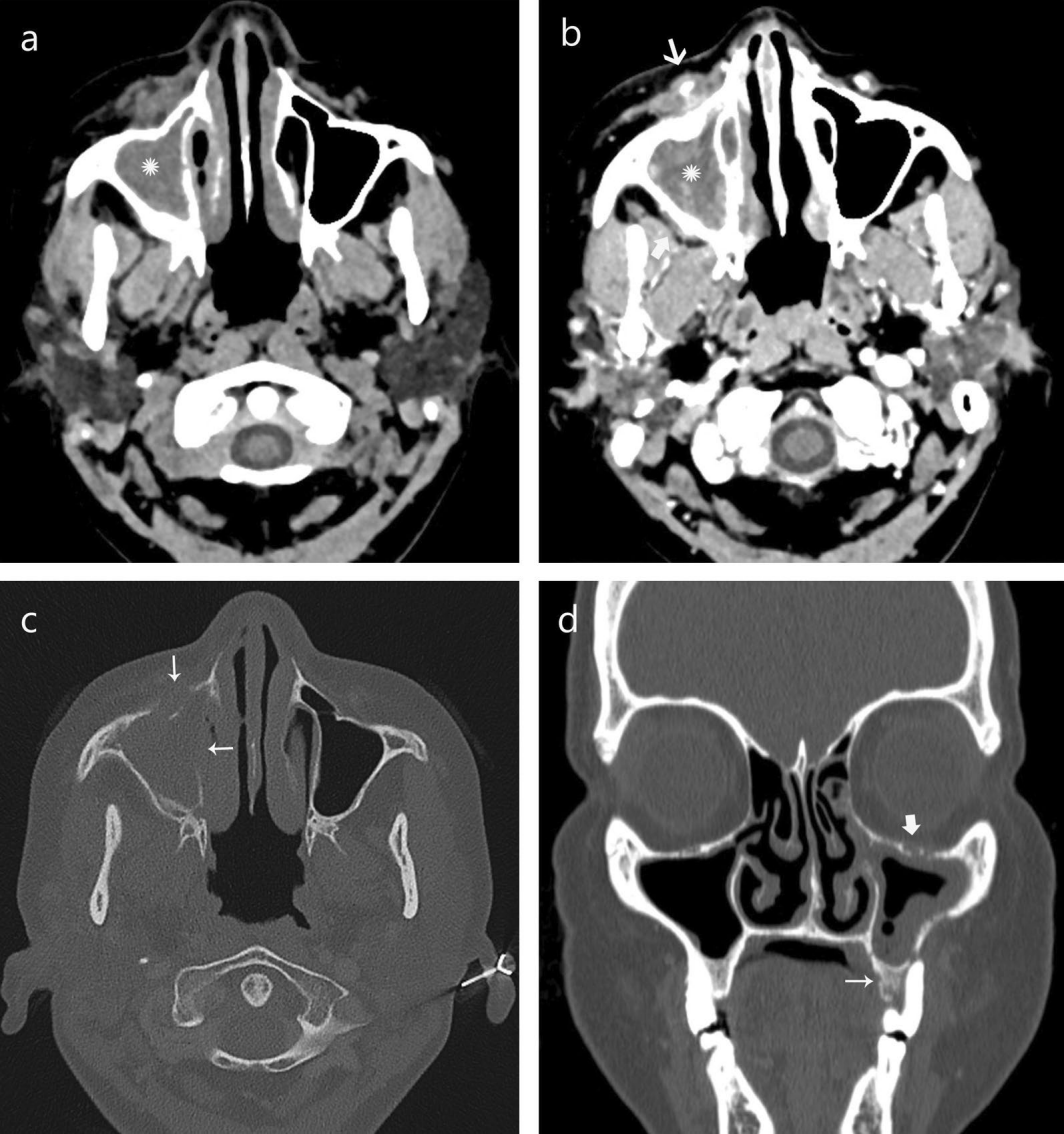
Computed tomography findings in mucormycosis. **a** Non-contrast and **b** contrast enhanced CT scan demonstrates hypodense soft tissue obliteration of right maxillary sinus (*) with heterogeneous, patchy mucosal enhancement and enhancing foci in cavity with inflammatory changes in premaxillary (thin arrow), retroantral fat (thick arrow) and facial muscles. **c** CT scan 20 days later shows extensive erosion of anterior and medial walls of maxillary antrum (arrows). **d** Coronal view of face shows mucosal thickening of left maxillary and anterior ethmoidal air cells, obliteration of the osteo-meatal complex, erosion of maxillary antrum (superior and lateral wall) and alveolar arch (thin arrow) along gingivo-buccal sulcus against 2^nd^ upper molar. The left infraorbital foramen is thickened suggesting perineural spread along infraorbital nerve (thick arrow)

1. *Sinonasal involvement*
  Eleven patients underwent CT examination (Table 2). All these patients had hypodense mucosal thickening in one or more sinuses. Four patients (36%) had hypodense sinus contents as well. None had intra-sinus hyperdensity (Fig. 1).

**Table 2:** Findings on CT imaging (*n* = 11)

CT findings	Attenuation	*n* = 11 (%)
Contents	Hypodense	4 (36)
	Hypodensity with hyperdense contents	0
Mucosal thickening	Hypodense	11 (100)
Enhancement	Mild heterogenous enhancement	6 (86)
	Intense enhancement	1 (14)

Seven of the CT examinations done were contrast enhanced studies. We identified two patterns of enhancement—mild heterogeneous enhancement seen in 6 cases (86%) and intense mucosal enhancement seen in 1 patient (14%). The presence of other concomitant findings in periantral space, orbit, brain and bone is shown in Table 1. These indicated the acute, invasive and aggressive nature of mucormycosis.

**Fig. 2:**
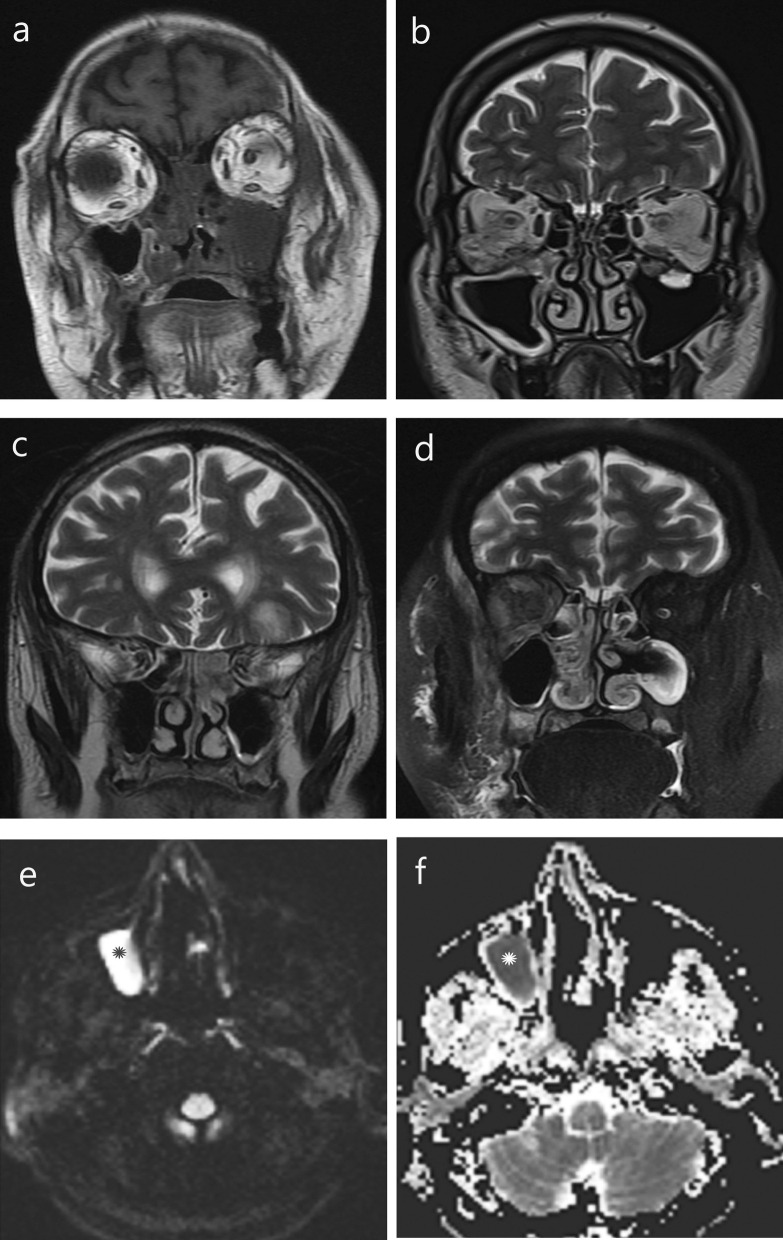
Magnetic resonance imaging findings in mucormycosis. **a** T1 weighted images showing hypointense polypoidal soft tissue filling bilateral ethmoids and left maxillary antrum with non-visualized left middle and inferior turbinates.T2 weighted images show **b** hyperintense mucosa involving bilateral maxillary sinuses with extensive inflammation in right intra- and extraconal orbital fat, **c** isointense soft tissue in bilateral ethmoids, **d** T2 fat suppressed images show hypointense mucosa in right ethmoid and nasal cavity, **e** Diffusion weighted images show high signal intensity in right maxillary sinus and **f** hypointensity in corresponding area (*) on ADC maps suggesting restricted diffusion

All the fourteen MRI studies were contrast enhanced (Table 3). Mucosal thickening was seen in 21 different sinuses in these patients. It was predominantly T1W hypointense or isointense to the adjacent muscles. T2W intensity patterns were variable as predominantly hyperintense in 8 (38%) sinuses, isointense or hypointense in 6 (29%) sinuses or predominantly hypointense in 7 (33%) sinuses. Intra-sinus contents were seen in 9 patients (64%). These were either hypointense on T1W and hyperintense on T2W (in 5 patients) or hypo/isointense on T1W and iso/hypointense on T2W (in 4 cases) (Fig. 2). One patient who presented with bloody nasal discharge had T1W hyperintensity lining the osteomeatal complex.

**Table 3:** MR Imaging findings (*n* = 21)

	T2 images	*n* = 21 (%)
Contents	Hypo	0
	Iso	4
	Hyper	5
Mucosa	Hypo	7 (33)
	Iso	6 (29)
	Hyper	8 (38)
Enhancement	Post-contrast T1 images	
	Intense homogenous	9 (43)
	Intense nodular/patchy	5 (24)
	Non-enhancing	7 (33)
Restricted diffusion		3

**Fig. 3:**
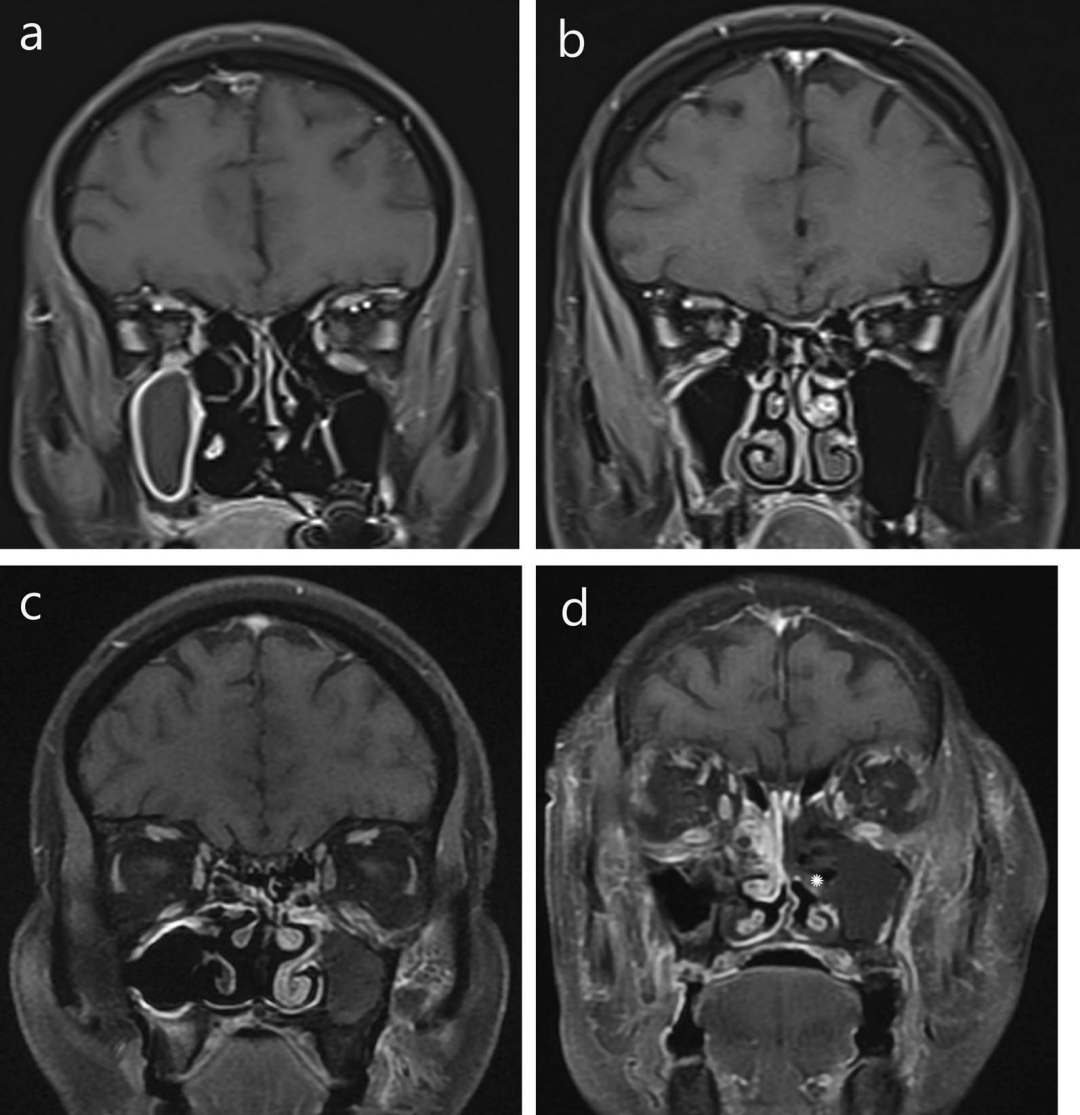
Mucosal enhancement patterns on MRI in mucormycosis. T1 contrast enhanced images in coronal view of face show **a** intense enhancement, **b** patchy heterogenous enhancement and **c** non-enhancing maxillary sinus mucosa, **d** non-enhancing left middle turbinate—“Black Turbinate sign” (*). Right ethmoidal air cells mucosa show intense enhancement. Extensive inflammation is seen in bilateral extraconal orbital fat, temporal and infra-temporal fossa

Mucosal enhancement was characteristically of three patterns—(1) intense homogenous enhancement in 9 sinuses (43%), (2) intense nodular/patchy enhancement in 5 sinuses (24%) and (3) no enhancement in 7 sinuses (33%). Non-enhancement was seen in T2W iso- or hypointense mucosa, suggesting necrosis. Black turbinate sign as described by Safder et al. [13] was seen in 5 patients (20%) (Fig. 3).

Out of five patients who underwent diffusion weighted imaging, three showed restricted diffusion in sinuses and nasal turbinates. These were the sinuses showing T2W iso- or hypointensity.

2. *Periantral involvement*
  This was seen in 18 patients (72%), suggesting highly specific finding of the disease (Table 4a). Most common involvement was retroantral fat streakiness seen in 16 cases (89%), followed by premaxillary fat streakiness in 14 patients (78%). Two patients had premaxillary abscess formation too. The inflammation was seen extending into adjacent buccal fat in 11 patients (61%), facial muscles in 7 patients (39%), pterygopalatine fossa in 6 patients (33%) and the masticator space in 10 patients (56%). Most common muscle involved was the medial pterygoid (80%). Extension of inflammation to the temporal fossa was seen in 44%, infratemporal fossa in 50% and palatal mucosa in 22% (Fig. 4). We also noted maxillary and mandibular alveolar arch involvement in 7 patients (39%). Sinus wall erosion was seen in only 2 cases (11%). Most of the above findings were seen in combination.

**Table 4:** Extra-nasal involvement

(a) Periantral involvement	*n* = 18 (%)
Premaxillary infiltration	16 (89)
Fat streakiness	14 (79)
Abscess	2 (11)
Buccal fat infiltration	11 (61)
Facial muscle involvement	7 (39)
Retroantral infiltration	16 (89)
Pterygopalatine fossa involvement	6 (33)
Masticator space involvement	10 (56)
Medial pterygoid	7
Lateral pterygoid	2
Masticator	1
Temporal fossa	8 (44)
Infratemporal fossa	9 (50)
Palate involvement	4 (22)
Maxillary alveolar arch	5 (28)
Mandibular alveolar arch	2 (11)
(b) Orbital involvement	*n* = 14 (%)
Proptosis	13 (93)
Preseptal space	12 (86)
NLD involvement	10 (71)
Extraconal fat	13 (93)
Streakiness	13
Subperiosteal collection	5
Intraconal space involvement	8 (57)
Ocular muscle involvement	
Medial rectus	5
Lateral rectus	3
Superior rectus	3
Inferior rectus	5
Superior oblique	3
Globe involvement	4 (29)
Optic nerve involvement	4 (29)
Stretched	3
Enhancement	1
Orbital apex	1(4)
(c) Intracranial extension	*n* = 5 (%)
Only meningeal enhancement	1 (20)
Infarct + meningeal enhancement	1 (20)
Abscess + meningeal enhancement	1 (20)
Extra axial collection	2 (40)
(d) Vascular involvement	*n* = 2
Cavernous sinus thrombosis	2
ICA thrombosis	2
(e) Bony changes	*n* = 8 (%)
Rarefaction	3 (37)
Erosion/destruction of	4 (50)
Cribriform plate	2
Medial wall of orbit	3
Inferior wall of orbit	1
Maxilla	4
Mandible	1
Antrum	1
Altered marrow signal intensity	1 (13)

**Fig. 4:**
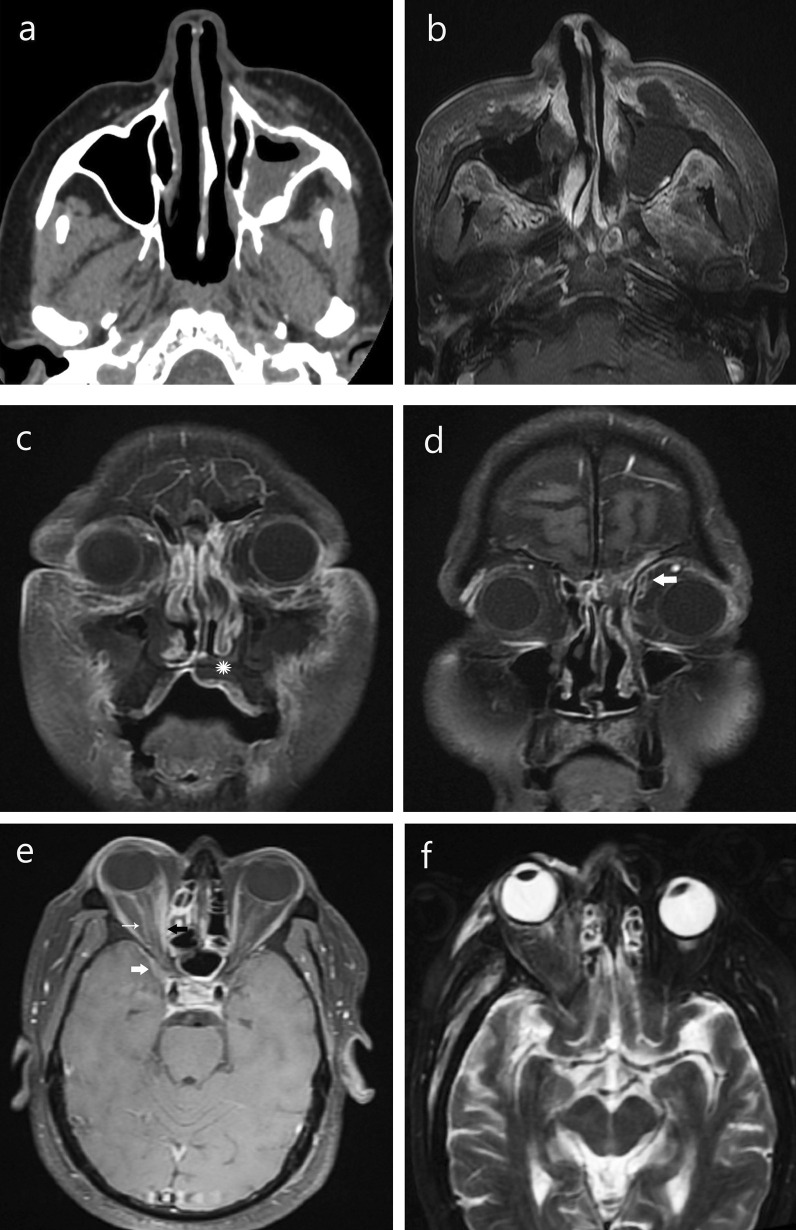
Imaging in early periantral inflammation. **a** Premaxillary and retroantral fat infiltration without bony erosion. **b** Bilateral premaxillary abscesses and intense enhancement of retroantral fat, masticator and infratemporal fossa with antral bony erosion. **c** Non-enhancing left half of anterior palate (*). **d** Early subperiosteal collection (arrow) and extraconal fat infiltration in supero-medial aspect of left orbit. **e** Enhancement of right optic nerve (thin white arrow), intraconal soft tissue extending till orbital apex (black arrow) and basitemporal meninges (thick white arrow). **f** Proptosis on right side with stretched optic nerve and distorted conical globe
3. *Orbital involvement*
  Fourteen patients (56%) showed features of orbital involvement (Table 4b). Proptosis and extra-conal fat streakiness were the most common findings, seen in 13 patients (93%). Small peripherally enhancing subperiosteal collections were seen in 5 patients (36%). In four patients (29%) who had complete ophthalmoplegia, three showed conical shape of globe with stretched optic nerve and one showed perineural enhancement along optic nerve extending till the orbital apex (Chandler grade 4 involvement) [7]. Another patient showed optic nerve involvement in repeat imaging done later in the course of disease, extending till optic chiasma.
4. *Intracranial and vascular extension*
  Five patients (20%) had intracranial extension of the disease process (Table 4c), seen as meningeal enhancement, basifrontal lobe abscess or ipsilateral cerebral infarcts due to internal carotid artery thrombosis. Two patients had cavernous sinus thrombosis which was seen as non-enhancing T2 hyperintense sinus (Table 4d; Fig. 5).
5. *Bony changes*

**Fig. 5:**
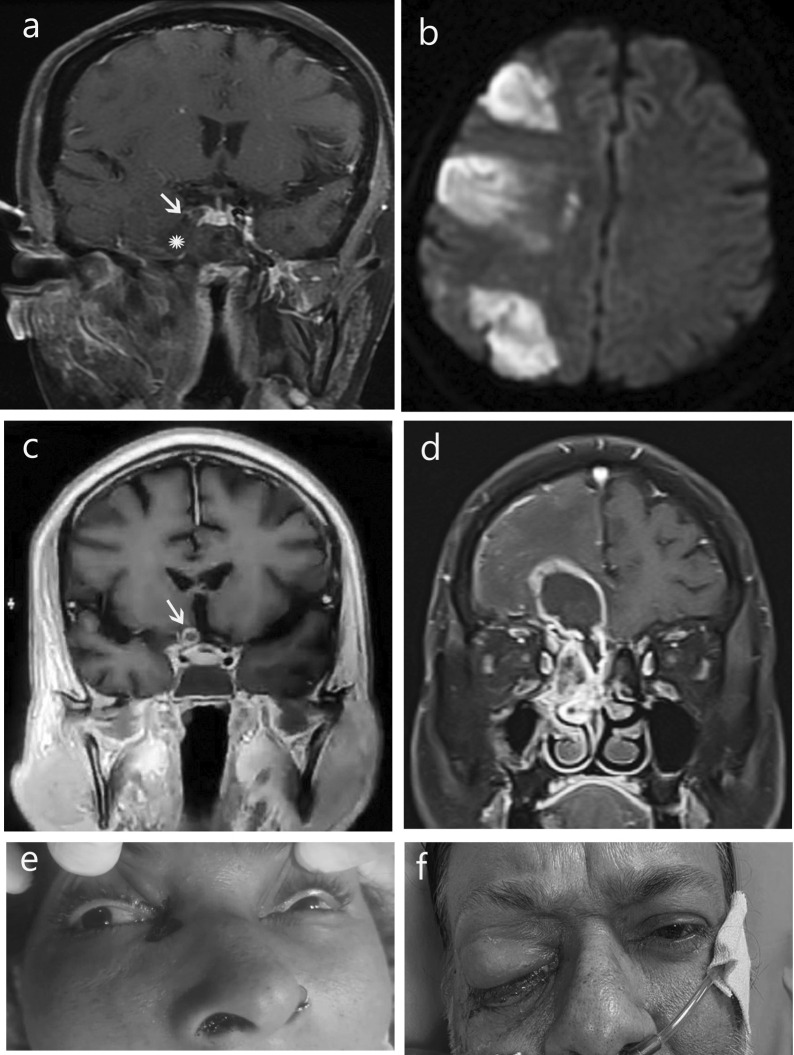
Intracerebral and vascular complications. **a** Non-enhancing soft tissue replacing right cavernous sinus (*) with loss of flow void in internal carotid artery (arrow), enhancing soft tissue inflammation in temporal and infratemporal fossa. **b** DWI showing restricted diffusion in multiple water shed infarcts. **c** Enhancing intracerebral optic nerve (arrow) on right side. **d** Cribriform plate erosion and ethmoidal mucosal thickening with basifrontal abscess. **e** Eschar at right medial canthus and nasal opening. **f** Proptosis and chemosis of right upper lid

Bony changes (Table 4e) were seen in 8 patients (32%). However, only 4 patients (50%) showed bony erosion or destruction, indicating subacute stage of infection. Rest showed rarefaction (3, 37%) or altered marrow signal (1, 13%) on MRI.

Interobserver agreement was calculated using Cohen’s Kappa, which was 0.0643 (Table 5).

**Table 5:** Contrast enhancement in sinuses on MRI interobserver variation

	Radiologist 1				
Radiologist 2	Type of enhancement	I	II	III	Total
	I	7	1	0	8
	II	2	4	2	8
	III	0	0	5	5
	Total	9	5	7	21

## Discussion

In our study, majority of the patients (60%) were between 40–60 years of age. Jacob Therakathu et al. [8] also reported 55% cases aged between 40–60 years. Sarkar et al. [9] and Mishra et al. [10] also reported 50% cases in the similar age group.

Acute invasive ROCM can progress in few hours to days with the fatality rate of 46% globally. With orbital and CNS involvement, it can be as high as 50–80% [11]. In a setting of COVID-19, Sarkar et al. and Mishra et al. have also reported 40% mortality [9, 10]. In our study, mortality was 24% (Table 6). These were the patients with intracranial involvement or extensive orbital involvement or bilateral sinonasal extension. The low mortality in our series may be the result of early detection of the disease due to high index of suspicion or already hospitalized status of the patients. Morbidity due to radical surgeries was significant. Imaging helps in early diagnosis, assessing the extent of involvement and diagnosing complications. This can help in guiding debridement and further medical and surgical planning.

**Table 6:** Early clinical management or outcome for ROCM (*n* = 25)

Management done	*n* = 25	Percentage (%)
Only I/V antifungals	2	8
Local debridement + Irrigation	5	20
Aggressive debridement + irrigation	2	8
Functional endoscopic sinus surgery	4	16
Exenteration	1	4
Rhinotomy + hemimaxillectomy	2	8
Death	6	24
Lost to follow-up	3	12

We noted the combination of maxillary, ethmoid and sphenoid sinuses involvement in 18 patients (72%), out of whom all had periantral involvement and 14 had concomitant orbital involvement. Som and Curtin [12] also found that concomitant sinonasal, periantral and orbital involvement with tissue necrosis was typical of acute invasive mucormycosis. Silvermann et al. [13] also indicated the aggressive nature of the infection by involvement of periantral and orbital fat.

CT scan shows findings of sino-nasal disease, mass effect, bony involvement and inflammatory changes in periantral tissues and orbit. However, abnormal mucosal enhancement patterns are difficult to demonstrate on CT. Contrast enhanced MRI is better at demonstrating early mucosal abnormalities, turbinate necrosis, non-enhancing devitalized tissues, orbital apex involvement and intra-cerebral extension.

In all the eleven patients, CT showed hypodense mucosa which showed intense or heterogenous mildly enhancing mucosal thickening, which was also reported by Herrere et al. [14] and Therakethu et al. [8]. In some patients, CT also showed bone rarefaction, erosions and permeative destruction. Bony erosion in medial wall of orbit or maxillary sinus wall was seen only in one case each. This can be explained by the fact that orbital extension also occurs through nasolacrimal duct, anterior and posterior ethmoid orifices or dehiscent medial orbital wall. Periantral spread can be due to perineural or perivascular extension of the fungal hyphae. Spread to brain occurs through the orbital apex, vessels or the cribriform plate of ethmoid bone, as seen in 2 out of 4 cases in our series [13, 14–16].

MRI showed T1 and T2 iso- or hypointense mucosa in sinuses in majority of the patients (72%). The signal intensity was more variable on T2W images with 33% sinuses showing hyperintensity. Variable signal intensity in MRI depends on the sinus contents due to iron and manganese in the fungal elements [17] and infarcted sloughed off mucosa due to fungal invasion of the adjacent blood vessels.

After the administration of gadolinium, the lesions showed three patterns of enhancement on MRI—intense homogenous enhancement in 43%, intense patchy enhancement in 24% and no enhancement in 33%. As a basic principle, increased tissue contrast enhancement in Invasive Fungal Rhinosinusitis implies active infection with inflammation, whereas loss of contrast enhancement signifies devitalization and necrosis [5]. Interobserver agreement using Cohen’s Kappa was 0.0643 suggestive of moderate–substantial agreement.

Restricted diffusion was seen in 3 out of 5 patients, who were assessed with diffusion weighted imaging. These were in the areas of non-enhancement. As the brain shows restricted diffusion in the areas of tissue ischemia and infarcted regions, the same can be related to the ischemia and necrosis resulting from fungal angioinvasion [18].

The main advantage of MRI is, however, the early identification of extra-sinonasal involvement and extension as also found by Raab et al. [19]. Orbital and vascular invasion was better delineated by MRI [20]. Seventy-two percent patients had periantral and 56% had orbital extension of the disease which was suggestive of acute invasive form of fungal infection. These findings, although subtle, pointed to an aggressive infective etiology rather than malignancy, given the short duration of history and immunocompromised status. Four out of 25 patients (16%) had palatal necrosis. Maxillary and mandibular alveolar erosions were also quite commonly seen in 5 (20%) and 2 patients (8%), respectively.

Involvement of the optic nerve, orbital apex, intracranial compartment and vascular structures was seen in 4%, 4%, 20% and 8% patients only. This could be attributed to high clinical suspicion and early imaging referrals due to outbreak of mucormycosis in Delhi and it being declared an epidemic. Also the patients were already under hospital care due to severe respiratory illness because of COVID-19.

Here, the differential diagnosis which could be considered includes bacterial infection/cellulitis, inflammatory pseudotumor, paranasal sinus tumor, Grave’s disease, carotico-cavernous fistula and cavernous sinus thrombosis. However, in bacterial etiology, blindness is a much later finding and early visual loss would favor the diagnosis of ROCM [21]. Also the rapidly progressive inflammatory changes without much bone involvement should suggest the suspicion of ROCM. The final diagnosis, however, rests on histopathology.

There is overexpression of inflammatory cytokines, impairment of cell-mediated immune response and decreased CD4 + T and CD8 + T cell counts in most of the COVID-19 patients, indicating their susceptibility to fungal co-infection [22]. Studies also show that SARS-CoV and SARS-CoV-2 belong to the same species and have the similar biological and clinical characteristics and prevalence [23]. Based on the studies on SARS in 2003, it was found that the incidence of fungal infection in SARS patients was 14.8–27% [24]. Also, fungal infection was accounted for the main cause of death in SARS patients, ranging up to 25–73.7% of death [25]. This makes it all the more important for early identification of concomitant fungal infections in patients suffering from COVID-19.

There were few limitations in our study. Firstly, we had a limited number of patients. Secondly, contrast enhanced MRI could not be performed in all the cases and diffusion weighted images were available in only five patients. Lastly, we had limited clinical and follow-up data available with us, as some of the patients had to be shifted elsewhere due to limited availability of hospital beds in India during the peak of the outbreak. So the predisposing risk factor assessment, further clinical treatment and outcome could not be studied.

## Conclusion

COVID-19 infection and its management make the patient susceptible to mucormycosis. Early diagnosis and prompt medical and surgical intervention is the mainstay of treatment. Early clinical signs are often underappreciated, but prompt imaging may help in timely diagnosis. Contrast enhanced CT and MRI are the imaging tools that can assess the complete extent of disease. MRI is better at demonstrating the extra-sinus, orbital, intracranial and vascular complications. Periantral infiltration and extraconal orbital extension even without bony erosion is a significant observation in acute invasive form. Palatal and alveolar arch involvement is also a common finding. Awareness and careful attention should be given to the subtle but often present radiological features of sinonasal mucosal enhancement. This along with combined demonstration of nasal, sinus, periantral and orbital disease allows the radiologist to help refine the diagnosis of acute invasive rhino-orbito-cerebral mucormycosis with more confidence in the clinical setting of COVID-19 and guide clinicians in further evaluation and treatment. This can greatly reduce the high morbidity and mortality associated with this disease.

## Supplementary Information

**Additional file 1.** Ethical Committee Guidelines.

## Data Availability

The masterchart is not being shared to safeguard patients’ privacy. However, we agree to share the same when specifically asked for.

## Abbreviations

ROCM: Rhino-orbito-cerebral mucormycosis
COVID-19: Corona virus disease-19
RT-PCR: Reverse transcriptase polymerase chain reaction
SARS-CoV2: Severe acute respiratory syndrome corona virus 2
CT: Computed tomography
MRI: Magnetic resonance imaging
