# Supplementary material for: A multicentric observational study of imaging findings in COVID-19-related rhino-orbito-cerebral mucormycosis: a new Pandora’s box
Source: Egypt J Radiol Nucl Med. 2021 Oct 20;52(1):258. doi: 10.1186/s43055-021-00631-w (PMC8527445; doi:10.1186/s43055-021-00631-w)
Supplement: Supplementary file 1 — Additional file 1. Ethical Committee Guidelines. [file 43055_2021_631_MOESM1_ESM.pdf]

NATIONAL GUIDELINES FOR ETHICS COMMITTEES REVIEWING BIOMEDICAL & HEALTH RESEARCH **DURING COVID-19 PANDEMIC**

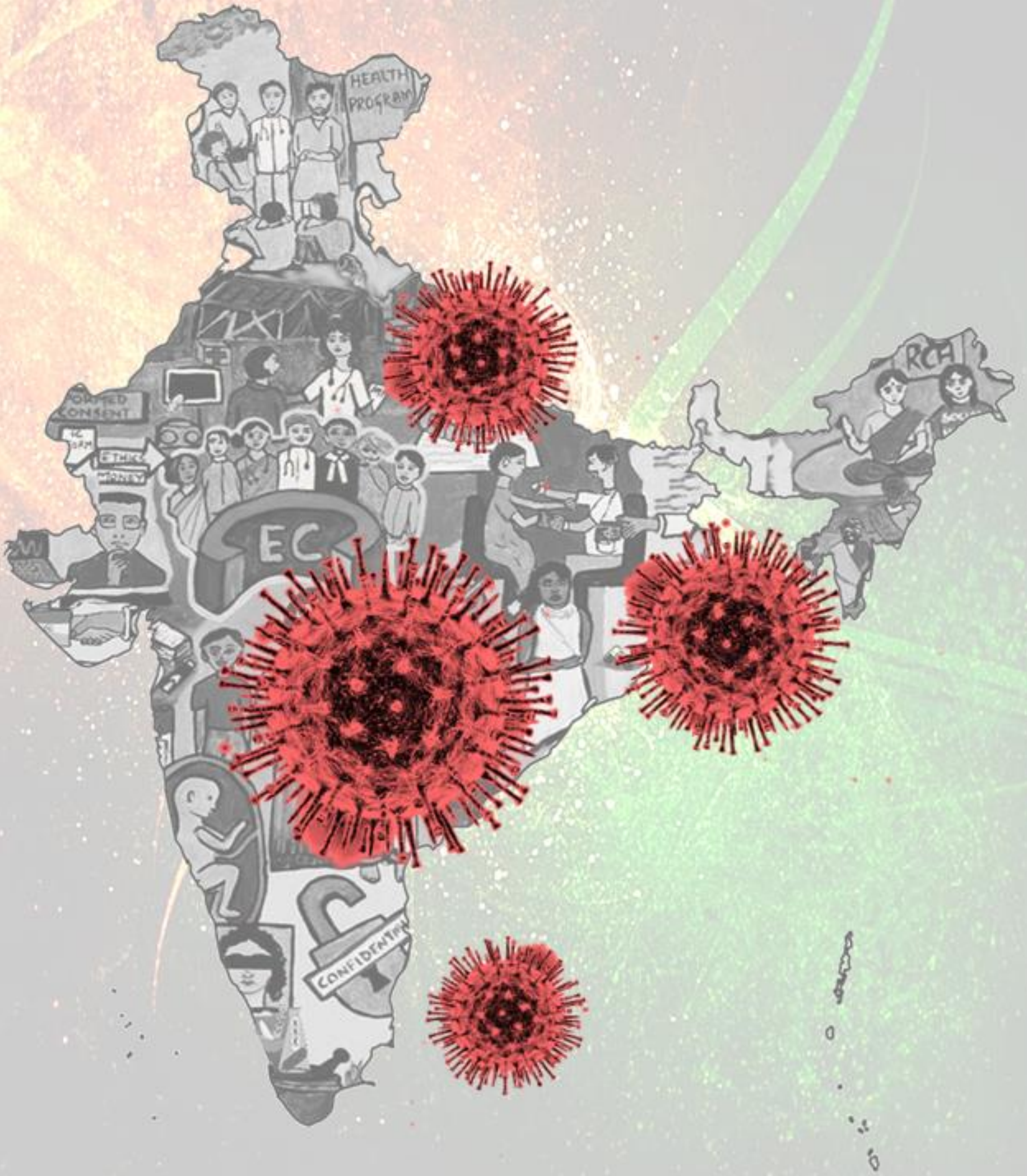

INDIAN COUNCIL OF MEDICAL RESEARCH

APRIL 2020

Compiled and Edited by:

Dr Roli Mathur, Scientist F & Head, ICMR Bioethics Unit, NCDIR, Bengaluru

Secretariat: Dr Kalyani Thakur & Dr Deepika Rathna M

Cover page design: Ms Ankita Pardhi

Published by:

ICMR - National Centre for Disease Informatics & Research, Bengaluru

for Director General, Indian Council of Medical Research, New Delhi

April 2020

Indian Council of Medical Research

Contents

Foreword ... **i**
Preface ... **ii**
1. Statement of General Principles ... **1**
2. General Ethical Issues ... **1**
2.1 Benefit-risk assessment ... *1*
2.2 Privacy Confidentiality ... *1*
2.3 Distributive Justice. ... *1*
2.4 Payment for participation. ... *1*
2.5 Compensation for research-related harm ... *1*
2.6 Conflict of interest ... *2*
2.7 Community Engagement ... *2*
2.8 Post research access and benefit sharing ... *2*
2.9 Storage of Biological Material/ datasets ... *2*
2.10 Collaboration in research ... *2*
2.11 Public health and socio behavioral research ... *3*
2.12 Role of Agencies/ Sponsors & Governance of Research ... *3*
2.13 Biosafety in laboratories and hospitals ... *4*
3. Ethical Review Procedures ... **4**
3.1 Categories of Research ... *4*
3.2 Ethics Committee (EC) ... *4*
3.3 Special Situations ... *5*
3.4 Ethics Review ... *5*
3.5 Review of Multicentre Research ... *6*
3.6 Continuing Review & Monitoring ... *6*
3.7 Decisions Regarding Ongoing Studies ... *6*
3.8 Review of new non-COVID Research ... *7*
4. Informed Consent ... **7**
4.1 Informed Consent Process ... *7*
4.2 Electronic Consent ... *8*
4.3 Waiver of Consent ... *8*
5. Vulnerability ... **8**
5.1 Vulnerable Persons ... *8*
5.2 Additional Safeguards ... *9*
5.3 Safety of Health Care Workers (HCW) involved in research ... *9*
5.4 Psychological needs and mental health ... *9*
References ... **9**
Annexure I: SOP Template for Ethics Review of during COVID-19 Pandemic ... **11**

सत्यमेव जयते

प्रोफेसर (डा.) बलराम भार्गव, पदम श्री

एमडी, डीएम, एफआरसीपी (जी.), एफआरसीपी (ई.), एफएसीसी,
एफएएचए, एफएएमएस, एफएनएएस, एफएएससी, एफ.एन.ए., डी.एस.सी.

सचिव, भारत सरकार
स्वास्थ्य अनुसंधान विभाग
स्वास्थ्य एवं परिवार कल्याण मंत्रालय एवं
महानिदेशक, आई सी एम आर

Prof. (Dr.) Balram Bhargava, Padma Shri

MD, DM, FRCP (Glasg.), FRCP (Edin.),
FACC, FAHA, FAMS, FNASc, FASc, FNA, DSc

Secretary to the Government of India
Department of Health Research
Ministry of Health & Family Welfare &
Director-General, ICMR

icmr
INDIAN COUNCIL OF MEDICAL RESEARCH
Serving the nation since 1911

भारतीय आयुर्विज्ञान अनुसंधान परिषद
स्वास्थ्य अनुसंधान विभाग
स्वास्थ्य एवं परिवार कल्याण मंत्रालय
भारत सरकार
वी. रामलिंगस्वामी भवन, अंसारी नगर
नई दिल्ली - 110 029

Indian Council of Medical Research
Department of Health Research
Ministry of Health & Family Welfare
Government of India
V. Ramalingaswami Bhawan, Ansari Nagar
New Delhi - 110 029

Foreword

A humanitarian emergency such as COVID-19 global pandemic has represented a critical threat to the health, safety, security and well-being of any community or large group of people across the globe. Research is necessary in such circumstances to enable provision of efficient and appropriate health and humanitarian response for reducing morbidity and mortality. In the present situation of lockdown, the health system, communications, research infrastructure, and research governance frameworks are adversely affected and have created challenges for the review, conduct and monitoring of research to safeguard the safety, rights and well-being of research participants.

On one hand, there is a need to undertake research quickly on priority basis, and on the other to ensure that this does not compromise scientific validity and ethical requirements. Close attention on perceptions of ethical questions, altered or increased vulnerabilities, health care provider–patient and researcher–participant relationships, issues related to integrity of studies and ethical review processes is needed. The complex issues that are raised need to be effectively and efficiently tackled.

This is the appropriate time to respond to humanitarian emergencies by adopting novel methodologies, designing innovative methods, appropriate use of digital platforms and new technology, effective management of limitations of time, infrastructure, resources and enable quick communication.

I am happy that ICMR Bioethics Unit, NCDIR, Bengaluru has developed this document under the guidance of Covid 19 National Ethics Committee (CoNEC) highlighting the important and facilitatory role that ethics committees will have to play in supporting the ethical conduct of research in India. They need to respond and adapt to the changing environment and to guide quality research outcomes in a time bound manner. I am sure all stakeholders in research such as researchers, sponsors, regulators etc will also find this document very useful in addition to Ethics committees.

(Balram Bhargava)

Tele.: 26588204, 26589620, Fax (Off.) : 91-11-26588662, E-mail: secy-dg@icmr.gov.in

Preface

Indian Council of Medical Research has always been on the forefront to develop ethical guidance for biomedical and health research in the country since 1980 and responded to ethical changes with the emerging times and ICMR National Ethical Guidelines is widely respected and recognised. In the ongoing COVID-19 pandemic situation, research has to take the front stage in order to tackle the novel challenges that have come to the fore in an unprecedented manner. There is need for extensive research to explore therapeutic options, deal with clinical challenges related to patient management and care, undertake epidemiological studies, fast track development of new diagnostic tools, identifying and tackling challenges impacting socio behavioural well-being, ways of reducing stigmatisation, need for quick research without compromising scientific integrity, sharing the samples, data transfer etc while protecting the rights, safety and well-being of research participants. The need for social distancing and nationwide lockdown has led to people from all strata of society being left in a vulnerable situation whether they are affected population, patients, family members, care givers or health care workers. Many have been forced to remain for long periods in isolation or quarantine, restricting their freedom of movement, psycho-social responses and autonomous decision making.

Section 12 on "Research during humanitarian emergencies and disasters" and several other sections of ICMR National Ethical Guidelines for Biomedical and Health Research Involving Human Participants, 2017 directly provide ethical guidance to conduct research in humanitarian emergencies. The relevant portions are being extracted and put together with additional new guidance points relevant to the COVID-19 situation in this document to facilitate easy understanding. The structure of the document is kept similar to the original guidelines for easy reference. Readers are encouraged to go through the full guidelines for details.

The role of ECs is very important in reviewing protocols prepared for such emergency situation(s). Responsiveness to the situation include, use of expedited or fast track processes but ensuring robust ethics review, as well as for monitoring conduct of research. During this pandemic and restricted environment of research, even non-COVID health research needs to be ongoing. There are provisions in the guidelines to facilitate them. It is expected that this guideline will be useful not only for ethics committees but for all stakeholders in research including researchers, sponsors and even public at large to inform them about the ethical conduct and review of research for ensuring participant safety and right at all time.

We are grateful to Prof. Balram Bhargava Secretary, DHR & DG, ICMR for his constant support and guidance. We thank the experts for reviewing the document and providing valuable inputs, viz., Dr NK Arora, Dr BT Kaul, Dr Bikash Medhi, Dr Shuba Kumar and Rv Dr Christopher Vimalraj. We deeply acknowledge the support extended by Dr Prashant Mathur, Director NCDIR, Bengaluru for fast track creation of this document. We also thank Dr Kalyani Thakur and Dr Deepika Rathna, staff of ICMR Bioethics Unit, NCDIR, Bengaluru for their assistance in developing this document.

Dr Vasantha Muthuswamy
Chairperson

Dr Roli Mathur
Member Secretary

1. Statement of General Principles

1.1 The four basic principles namely; **respect for persons (autonomy), beneficence, non-maleficence and justice** must guide research in order to protect the dignity, rights, safety and well-being of research participants while conducting Biomedical and Health Research.

1.2 These basic principles have been further expanded into 12 general principles:

i.	Principle of Essentiality	ii.	Principle of Professional Competence
iii.	Principle of Voluntariness	iv.	Principle of Maximization of Benefit
v.	Principle of Non-exploitation	vi.	Principle of Institutional Arrangements
vii.	Principle of Social Responsibility	iii.	Principle of Transparency & Accountability
ix.	Principle of Ensuring Privacy & Confidentiality	x.	Principle of Totality of Responsibility
xi.	Principle of Risk Minimization	xii.	Principle of Environmental Protection

2. General Ethical Issues

2.1 **Benefit-risk assessment:** The EC must decide about the type of review required (exempted, expedited, full committee) based on the type of risk involved.

Table 1: Types of risk

Type of risk	Definition/description
**Less than minimal**	Probability of harm or discomfort is nil or not expected.
**Minimal risk**	Probability of harm or discomfort anticipated in the research is not greater than encountered in routine life activities/ serious harm or adverse event is unlikely
**Minor increase over minimal risk or Low risk**	Increment in probability of harm or discomfort is only a little more than the minimal risk threshold. Such research should have a social value. Social risks, psychological harm and discomfort may also fall in this category.
**More than Minimal/high risk**	Probability of harm or discomfort anticipated in the research is invasive and greater than minimal risk or interventional study.

2.2 **Privacy Confidentiality:** Information related to COVID-19 infection may be highly sensitive in nature with a lot of scope for stigmatization, discrimination, violence etc. Maintaining confidentiality of research related data and its publication is important to protect the privacy of individuals and avoid any discrimination against them.

2.3 **Distributive Justice:** Individuals or communities invited for research should be selected in such a way that the benefits and burdens of research are equitably distributed without leading to social, racial or ethnic inequalities.

2.4 **Payment for participation:** Participants should not be made to pay for any expenses incurred beyond routine clinical care and wherever possible may be given a reasonable amount to cover incidental expenses.

2.5 **Compensation for research-related harm:**

2.5.1 Research participants who suffer direct physical, psychological, social, legal or economic harm as a result of participating in the research are entitled to free health care and referrals as needed. However, for research related Serious Adverse Events (SAE), appropriate financial compensation and insurance coverage be provided as per norms.

2.5.2 Sponsor to include insurance coverage/other provision within budget. In investigator initiated research, investigator/institution must provide through insurance, corpus funds or grants.

2.5.3 SAEs should be reported to EC (including on non-working days) within 24 hours and a report on SAE relatedness (causality assessment) within 14 days for EC review regarding quantum and type of assistance.

2.6 Conflict of interest:

2.6.1 Implement procedures to declare and management conflict of interest (financial/non-financial) of researchers, EC members, institution, sponsor.

2.7 Community Engagement:

2.7.1 Engaging with the community in a culturally sensitive manner can improve public trust, help improve design, conduct and responsiveness to health needs.

2.7.2 Various measures to educate the public or communities about pandemic (COVID-19 infection), proposed research, risks and benefits, persons to be contacted etc. should be undertaken.

2.7.3 Efforts must also be made to prevent the infodemic or spread of fake information, or to sensationalize, make false promises or claims, or spread negativity or create a scare.

2.7.4 Wherever possible, community representatives (e.g., Community advisory board) be involved in conceptualization, review, research, dissemination of results in such settings.

2.8 Post research access and benefit sharing:

2.8.1 Efforts be made to communicate the research findings to the individuals/communities.

2.8.2 EC should consider the need for an a priori agreement between researchers and sponsors regarding post-research access of the community to successful interventions and benefit sharing if relevant.

2.9 Storage of Biological Material/ datasets:

2.9.1 In COVID-19, samples may be in the form of expectorated sputum, endotracheal aspirate, or Broncho alveolar lavage (BAL) etc. besides other body fluids, such as blood, plasma, dried blood spots, body fluids, urine, stool, tissues, organs - stored or prospectively collected. Storage of infectious samples requires adequate safeguards.

2.9.2 A dataset is an organized collection of data and information maintained in physical and/or electronic/digital form ranging from small numbers to large numbers or whole population.

2.9.3 Samples/ datasets may be classified as anonymous (unidentified), anonymized or identifiable.

2.9.4 It may be useful to have repository of samples/ registry which can be further used to generate forecasts of trends and identify hotspots.

2.9.5 Provide clarity on custodianship, obtain approval of the EC/ governance committee, appropriate written consent, maintain individual confidentiality and privacy.

2.10 Collaboration in research:

2.10.1 Existing guidelines on collaboration for sharing biological samples, data and intellectual property including publication related issues will be applicable. Collaborations to address possible inequity of expertise, access between partnering institutions /funding relationships.

2.10.2 Rapid data sharing while safeguarding the above is critical during public health emergency.

2.10.3 An appropriate MoU and/or MTA to safeguard the interests of participants and ensure compliance (addressing issues of confidentiality, sharing of data, joint publications, etc).

2.10.4 Biomedical and health research proposals involving foreign assistance and/or collaboration be submitted to Health Ministry’s Screening Committee (HMSC) for approval before initiation.

2.11 Public health and socio behavioral research:

2.11.1 Health system preparedness is critical to control spread of COVID-19 and focused research and public health interventions are needed to prevent, delay, or contain the spread.

2.11.2 Isolation, quarantine, segregation from families during the COVID-19 disaster has given a new dimension to risk to individual dignity, psychological/ emotional harm, social harm, informational risk.

2.11.3 Emergency circumstances have rendered participants vulnerable to be coerced to participate. They may not have access to formal or informal support during these times e.g. families, counselling centers, rehabilitation centers, police protection, etc.

2.11.4 The social distancing norms may not facilitate conventional methods of data collection and alternative study designs may be required such as online or remote methods to conduct interviews, focus groups, surveys or questionnaires. Social media research using data in public domain may still be evaluated for potential privacy threats.

2.11.5 Stakeholders are to consider the fact that technological requirements of the study design may exclude participants without access to the technology.

2.11.6 For obtaining quality data, verification of identity of research participant is required. However, exchanging confidential information electronically is prone to security threats. The privacy and security features of the virtual tool used must be assessed to a reasonable extent.

2.11.7 Collection of identifying information, GPS location, IP address tracking, etc. should be reviewed by EC on case-case basis.

2.12 Role of Agencies/ Sponsors & Governance of Research:

2.12.1 Humanitarian emergencies lead to fragile political environments, disruption of health systems, challenging social situations, resource constraints for fast track conduct of research.

2.12.2 Need to ensure appropriate safety, funds, care and compensation, including insurance coverage as well as training at individual, societal and/or community levels for patients, health care workers and others engaged in COVID-19 research.

2.12.3 Setting up community consultations and preparation of public educational material.

2.12.4 Support for extensive expert group consultations, review of existing national and international experience, adequacy of data from preclinical or previous clinical evidence, public private involvement, ensuring best minds get together to guide research in-spite of limited scientific evidence, ensure robust research protocols and outcomes.

2.12.5 Central regulatory authority to undertake expeditious review process for clinical trials for new drugs/ compassionate use and ensure safety/efficacy monitoring processes.

2.12.6 In case of an outbreak of infectious diseases, monitored emergency use of unregistered and experimental interventions (MEURI) may be approved with the following precautions:

- Thorough scientific review followed by an ethics review / locally or by national level EC
- Tackle public concerns and ensure oversight by a local EC.
- Use GMP products, make rescue medicines/supportive treatment accessible.
- Meticulous documentation of therapeutic processes including adverse events
- Fast track research and possible sharing of data on safety and efficacy for further research
- Consent process is important and must be carried out with care.
- Community engagement and ensuring fair distribution of scarce supply

2.12.7 Facilitate post-trial access of the successful investigational drug/ vaccine free of cost to the trial participants till the same is available in the market.

2.12.8 Media must also play a responsible role in facilitating dissemination of useful information and not creating panic through spread of unauthenticated information.

2.13 Biosafety in laboratories and hospitals:

2.13.1 There are four biosafety levels from BSL-1 to BSL-4 with specific controls for containment of microbes and biological agents. Virus isolation in cell culture and initial characterization of infectious viral particles recovered in cultures of SARS-CoV-2 specimens should only be conducted in a Biosafety Level 3 (BSL-3) laboratory or BSL-4 laboratories which offer highest safety environments.

2.13.2 The lab must ensure proper labelling and handling of specimens (suspected or confirmed for COVID-19) and relevant biosafety precautions and relevant regulatory standards to protect individuals and the environment/ testing in National Accreditation Board for Testing & Calibration Laboratories (NABL) certified labs. Regulatory requirements for biosafety labs should be strictly followed as prescribed by Department of Biotechnology (DBT) and Min. of Environment and Forests, Govt. of India.

2.13.3 Personnel must be trained about additional precautions, decontamination with appropriate disinfectants, hand hygiene, use of personal protective equipment (PPE), or other physical barriers, biomedical waste handling to reduce the risk of exposure.

2.13.4 Every effort should be made to limit contact with patients at triage, cohort of patients with COVID-19, limit the numbers of staff providing care.

2.13.5 Ensure that active screening of all staff at the hospitals is done daily and implement cleaning and disinfection protocol.

2.13.6 Telemedicine can be used for research when possible. Patient consent is necessary for any telemedicine consultation for research.

3. Ethical Review Procedures

3.1 Categories of Research:

3.1.1 There are 3 categories of research during COVID that may require ethics review.

- New research directly related to COVID-19
- Ongoing non-COVID research
- New non-COVID research

3.1.2 EC must prioritize research review based on urgency and take needful steps to facilitate the review of new research and conduct ongoing research with needful amendments as per need in the view of social distancing norms.

3.2 Ethics Committee (EC):

3.2.1 EC to ensure a thorough scientific and ethical review of research as per national guidelines and regulations to safeguard the dignity, rights, safety and well-being of research participants.

3.2.2 EC to be registered with appropriate agencies – DHR for biomedical and health research and CDSCO for regulatory clinical trials as per New Drug and Clinical Trial Rules, 2019.

3.2.3 EC to ensure that all COVID-19 related research (all clinical trials as well as biomedical and health research) be registered on Clinical Trial Registry of India (CTRI) and seek approvals as per relevant guidelines and applicable regulations.

3.2.4 Member Secretary to categorise proposals into exempt/expedited/ or full review category as per National Ethical Guidelines and plan next steps for fast track review.

3.2.5 Research during emergencies can be reviewed through expedited review/unscheduled full committee meetings on a case-to-case basis depending on the urgency and need. If an expedited review is done, full ethical review can follow whenever next possible.

3.2.6 Quorum for decision-making should have a minimum of five members, including both medical/non-medical or technical/non-technical members with one non-affiliated member.

3.2.7 Measures such as virtual or tele/web conferences should be attempted and face-to-face meetings can be avoided to observe social distancing norms.

3.2.8 In exceptional and emergency situations, preliminary research procedures including but not restricted to data/ biological sample collection that are likely to rapidly deteriorate or perish may be allowed while the ethics review process is still underway.

3.2.9 Available protocol templates could be reviewed to expedite the process and interim review/ re-review can be done if the emergency situation changes.

3.2.10 In situations where members of local ECs are unavailable, the review may be conducted by any other EC within India for initiating the study, until the local EC is able to convene its meeting.

3.2.11 ECs should develop procedures to ensure timely review and monitoring of the approved research. On a case-by-case basis, may require re-review with time and circumstances.

Table 2: Ethical issues related to reviewing a protocol

Social values	Scientific design and conduct of study	Review of informed consent process
Benefit–risk assessment	Selection and recruitment of participants	Qualification & adequacy of study sites
Payment for participation	Disclosure of conflict of interest	Plans for medical management and compensation for study related injury
Community considerations	Protection of privacy and confidentiality	

3.3 Special Situations:

3.3.1 Institutions can have multiple ECs as per need or may utilize services of another institution with mutual agreement and agree to be overseen by it.

3.3.2 Registered Independent ECs (Ind EC) can review protocols of researchers who have no institutional attachments or of institutions without their own ethics committees.

3.3.3 Institutions could have subcommittees such as SAE subcommittee or expedited review committee which report to the main EC. These comprise Chairperson/ Member Secretary and one to two designated members of the main EC as defined in the SOPs.

3.4 Ethics Review:

3.4.1 Researchers should submit research proposals in the ICMR Common Forms for Ethics Review as soft or hard copies enclosing required documents.
(http://ethics.ncdirindia.org/Common_forms_for_Ethics_Committee.aspx)

3.4.2 The EC should adopt/ include an SOP for Emergency Research review.

3.4.3 Submission of e-copy of research protocol and relevant documents followed by their screening by Secretariat for completeness and categorization as exempt/ expedited review/ emergency full committee review depending on the urgency and need.

3.4.4 Electronic documents may be accepted for review and timelines shortened for accelerated procedures.

3.4.5 Virtual or Tele/Video conferences should be attempted to ensure social distancing as face-to face meetings may not be suitable. Use suitable virtual software platform, preferably a video conference to enable face to face discussion or teleconference if connectivity is an issue.

3.4.6 Agenda of virtual meetings should be kept short, however, EC may meet more frequently for fast track review within in 24-48 hrs.

3.4.7 The EC may plan a prior review by subject experts/obtain clarifications from researchers before the meeting or/ invite independent consultants (non-voting) or representative from a

specific patient group as special invitee. The special invitees invited for the web-meeting may be asked to leave the meeting before final decision making.

3.4.8 During the review process, the Ethics Committees should consider the following:

- If written consent is not possible (e.g., physical isolation/severe COVID-19 patients), consent could be given orally/ use electronic methods to document and record.
- Due to inability of the participant to attend the site (for e.g., social distancing), the contact/communication can be made via phone, to enquire and identify adverse events, serious adverse events and ensure medical care and oversight with documentation.
- In an ongoing study, if the designated principal investigator (PI) is indisposed for a period, she/he may need delegate parts of her/his duties temporarily to others/ co-investigator and the same should be documented and reported to EC at the earliest.

3.4.9 Withholding information in Public Health emergencies may be a threat to national security, and therefore the right balance must be maintained to protect individual privacy and confidentiality, and relevant disclosure to public health authorities.

3.4.10 Suggest steps to protect participants of researchers from possible stigma or discrimination.

3.4.11 EC members present during the virtual meeting should decide through consensus or cast online vote expressing their decision. Any disagreement to be recorded with reasons.

3.4.12 Meeting could be digitally recorded (audio/video) with permission of members and secretariat is responsible to note the attendance/ participation in the online meeting.

3.5 Review of Multicentre Research:

3.5.1 Common review of multicentre research in India can be carried out by one main designated EC for fast track decision making.

3.5.2 The local ECs are free to accept the decision of designated committee or to do an expedited or full committee review expeditiously. They must ensure ethics review of local site specific issues or concerns, informed consent translations, local study implementation and monitoring.

3.5.3 Common review is generally carried out for research involving low or minimal risk, survey or multicentric studies using anonymized samples or data or those that are public health research studies determined to have low or minimal risk.

3.5.4 However, in an emergency situation like the current one, for all types of research including high risk studies or those involving vulnerable population can be taken up for fast track common review while ensuring strict monitoring and oversight by registered local ethics committees.

3.6 Continuing Review & Monitoring:

3.6.1 The EC should continually evaluate progress of ongoing proposals, monitor approved study site for compliance, review SAE reports, protocol deviations/violations/ non-compliance/ DSMB reports/ any new information/assess final reports.

3.6.2 For protocol deviations/violations the EC should examine the corrective actions. If the violations are serious the EC may halt the study.

3.6.3 Compensation must be given for research-related injuries if applicable, as determined by the EC and as per regulatory requirement (if applicable).

3.7 Decisions Regarding Ongoing Studies:

3.7.1 The impact of COVID-19 on ongoing and existing studies, ongoing recruitment and continued involvement of participants needs to be considered.

3.7.2 Secretariat in consultation with Chairperson, must carefully evaluate need for other non COVID-19 research studies that are ongoing/ near term/ have direct benefit(s) and if stopped, may pose risk to participants. These may be continued/suggest mechanisms for continuation.

3.7.3 Following measures can be taken in consideration such as, extension of study duration; temporary halt of study at some/all sites; Suspension/ Postponement of study or activation of sites that have not yet been initiated without compromising safety and well-being of patients; Continuation of study with limited parameters; conversion of physical visits into phone or video visits, postponement or complete cancellation of visits to ensure that only strictly necessary visits are performed at sites; ongoing study may need to take re-consent of already enrolled participants to implement urgent changes; it can be done via phone or video-calls and obtaining oral consents supplemented with email confirmation.

3.7.4 Further, travel restrictions, confinement of study participants and staff to perform visits should be taken into account.

3.8 Review of new non-COVID Research:

3.8.1 If priority for ethics review in a defined timeframe is given to COVID-19 related research, non-COVID research must not suffer due to *'covidisation'*. Studies evaluating treatments for chronic conditions or other communicable diseases or injuries or others may also be considered for review by EC as these may also be important.

3.8.2 EC should review and assess if a planned study may have a negative impact on participants' safety or increase risk to participants (as a result of the ongoing COVID-19 pandemic), and make a decision to allow or not allow it so. It may also make relevant suggestions for additional safeguards for conducting research in such emergency.

3.8.3 The review of these studies may be done through virtual EC meeting ensuring appropriate scientific and ethical review and fulfilling the quorum requirements.

4. Informed Consent

4.1 Informed Consent Process:

4.1.1 Obtaining valid informed consent in humanitarian emergencies such as COVID-19 is a challenge due to practical difficulties in reaching out to a patient, who may be in a COVID ward, isolation or quarantine facility. In addition, the decisional capacity of the hospitalised patient with moderate or critical disease condition would be very low and it may not be possible to differentiate between reliefs offered and research components.

4.1.2 Informed consent is a continuous process involving three main components – providing relevant information, ensuring competence, ensuring comprehension and voluntariness.

Table 3: Elements of an ICD

Elements of an ICD	Additional elements (optional)
1. Statement mentioning that it is research	1. Alternative procedures or treatment
2. Purpose of research and methods	2. Insurance coverage
3. Duration, frequency, methods	3. Possible stigmatizing condition
4. Benefits to participant, community or others	4. Biological material and data, including
5. Foreseeable risks, discomfort or inconvenience	i. Current and future uses
6. Confidentiality of records	ii. Period of storage, secondary use, sharing
7. Payment/reimbursement for participation	iii. Right to prevent use of biological sample
8. Treatment and/or compensation for injury	iv. Provisions to safeguard confidentiality
9. Freedom to participate/withdraw	v. Post-research plan/benefit sharing
10. Identity of research team and contact persons	vi. Publication plan/photographs/pedigrees

4.1.3 Needful procedure be followed as discussed in National ethical guidelines for involving children (assent) or legally authorized representative (LAR) in case a participant is incompetent (medically or legally), illiterate participant/LAR should be witnessed by an impartial literate witness.

4.1.4 Broad consent with an individual informed opt-out option may be used for research on residual clinical samples.

4.1.5 The Informed Consent Document (ICD) has two parts – patient/participant information sheet (PIS) and the informed consent form (ICF) and can be prepared preferably utilizing electronic formats or plan methods to obtain consent maintaining adequate social distancing.

4.2 Electronic Consent:

4.2.1 In light of COVID-19 infection control measures, the alternative procedures to avoid direct interaction with the patient in isolation must be explored.

4.2.2 Technology should be utilized to prepare interactive formats and using electronic tools such as text, graphics, audio, video, podcasts, interactive website, platforms to explain information related to a study and to electronically document informed assent/consent the same.

4.2.3 Electronic methods (e.g. digital signature) must be reviewed and approved by the EC a priori.

4.2.4 Process can be documented through audio or video recording (if required).

4.3 Waiver of Consent:

4.3.1 For seeking waiver of consent, the researchers should give the rationale justifying the waiver which EC can approve a waiver after careful discussion in the following situations:

- research cannot practically be carried out without the waiver and the waiver is scientifically justified like, cluster randomization trials.
- retrospective studies, where the participants are de-identified or cannot be contacted
- research on anonymized biological samples/data
- certain types of public health studies/surveillance programs/program evaluation studies
- research on data available in the public domain; or
- research during humanitarian emergencies and disasters, when the participant may not be in a position to give consent.
- When consent of the participant/LAR/assent is not possible due to the emergency situation, informed consent can be administered at a later stage, when the situation allows for it, and if it is so envisaged, prior permission must be obtained from the EC.

5. Vulnerability

5.1 Vulnerable Persons are individuals/ belonging to certain groups of persons who are relatively or absolutely incapable of protecting their own interests such as:

5.1.1 COVID-19 patients may be additionally vulnerable of being stigmatized due to the contagious nature of the disease. Also at risk are health care workers in COVID-19 hospitals including doctors, nurses, ward staff, sanitation workers, security personnel, food suppliers, or others.

5.1.2 Socially, economically or politically disadvantaged individuals such as the stranded migrant workers who are susceptible to being exploited;

5.1.3 Incapable of making a voluntary informed decision or whose autonomy is compromised temporarily or permanently;

5.1.4 Able to give consent, but voluntariness/understanding compromised due to their situation;

5.1.5 Unduly influenced either by the expectation of benefits or fear of retaliation in case of refusal to participate which may lead them to give consent

5.1.6 Terminally ill patients ready to consent in search of new interventions.

5.2 Additional Safeguards: Participants may be under duress and traumatized, therefore, additional safeguards are required for participants and it should be ensured that,

5.2.1 Research to address the needs of participants and justify inclusion of vulnerable persons.

5.2.2 Benefits and risks carefully determined and the risk minimization strategies are examined.

5.2.3 There is no coercion, force, undue influence, threat or misrepresentation or incentives.

5.2.4 Informed consent process is conducted in a respectful manner.

5.2.5 Efforts to set up support systems to deal with associated medical and social problems.

5.2.6 Protection of their privacy, confidentiality and rights is required at all times.

5.2.7 Whenever possible, ancillary care may be provided.

5.3 Safety of Health Care Workers (HCW) involved in research:

5.3.1 In wake of the pandemic, safety of researchers must get due attention as transmission of infection to one member in a lab or clinical setting could jeopardize the entire program.

5.3.2 Ensuring safety is the responsibility of the institution, sponsors and local authorities, since research team may be subjected to disturbing instances (trauma, humiliation and threats of violence) while conducting research.

5.3.3 Additional precautions such as; Prioritize research and schedules to prevent overcrowding, adequate training, appropriate biosafety precautions, expose minimum number of researchers, communication using electronic platforms, due protection gear/PPE and facilities to undertake research, safety against any assault from public or others, insurance cover etc.

5.4 Psychological needs and mental health:

5.4.1 Persons tested positive for COVID-19, their families, health workers who get in contact with COVID positive cases must be provided due psychosocial support wherever possible.

5.4.2 There is need to show respect, empathy and compassion and not subject them to any kind of stigma or discrimination.

5.4.3 Persons in isolation or quarantine may face enormous stress and anxiety. Managing the mental health and psychosocial well-being is important.

5.4.4 The institutions must ensure access to psychosocial and emotional support, good communication, flexible working hours, and ways to ensure physical as well as psychological well-being and mental health of those going through the crisis.

References

1. National Ethical Guidelines for Biomedical and Health Research Involving Human Participants. New Delhi: Indian Council of Medical Research; 2017. Available from: (http://ethics.ncdirindia.org//asset/pdf/ICMR_National_Ethical_Guidelines.pdf). (Last accessed on 28th April 2020)
2. ICMR Common Forms for Ethics Review. Available from: (http://ethics.ncdirindia.org/Common_forms_for_Ethics_Committee.aspx). (Last accessed on 28th April 2020)
3. Central Drugs Standard Control Organization (CDSCO), Directorate General of Health Services Ministry of Health & Family Welfare, Government of India, New Delhi. Available from: https://cdsco.gov.in/opencms/opencms/en/Home/ (Last accessed on 28th April 2020)
4. New Drugs and Clinical Trials Rules 2019 G.S.R. 227(E). CDSCO. Available from: https://cdsco.gov.in/opencms/opencms/system/modules/CDSCO.WEB/elements/download_file_division.jsp?num_id=NDI2MQ==. (Last accessed on 28th April 2020)
5. National Accreditation Board for Hospitals & Healthcare Providers (NABH), Information Brochure for Ethics Committee Accreditation Program, Dec 2016 Available from: https://www.nabh.co/Images/PDF/CT_Brochure.pdf (Last accessed on 28th April 2020)

6. Clinical Trial Registry-India (CTRI), ICMR National Institute of Medical Statistics. Available from: http://ctri.nic.in/Clinicaltrials/login.php (Last accessed on 28th April 2020)
7. Department of Health Research (DHR), MOHFW, Government of India, National Ethics Committee Registry for Biomedical and Health Research (NECRBHR). Available from https://naitik.gov.in/DHR/Homepage (Last accessed on 28th April 2020)
8. Matt Richtel San Fransisco W.H.O. Fights a Pandemic Besides Coronavirus: an 'Infodemic' Available from: https://www.nytimes.com/2020/02/06/health/coronavirus-misinformation-social-media.html (Last accessed on 28th April 2020)
9. Guidelines for International Collaboration /Research Projects in Health Research; MoUs & HMSC procedure Available from: https://www.icmr.nic.in/content/guidelines (Last accessed on 28th April 2020)
10. ICMR Bioethics Unit NCDIR, National Centre for Disease Informatics and Research (Indian Council of Medical Research), Bangalore. Available from: http://ethics.ncdirindia.org/ (Last accessed on 28th April 2020).
11. UNESCO. Ethics in research in times of pandemic COVID-19, dated 26th March 2020 Available from: https://en.unesco.org/news/ethics-research-times-pandemic-covid-19. (Last accessed on 28th April 2020).
12. World Health Organization Ethical standards for research during public health emergencies: distilling existing guidance to support COVID-19 R&D. 21st March 2020. https://apps.who.int/iris/bitstream/handle/10665/331507/WHO-RFH-20.1-eng.pdf?sequence=1&isAllowed=y (Last accessed on 28th April 2020)
13. World Health Organization. (2016). Guidance for Managing Ethical Issues in Infectious Disease Outbreaks. https://apps.who.int/iris/bitstream/handle/10665/250580/9789241549837-eng.pdf?sequence=1&isAllowed=y (Last accessed on 28th April 2020).
14. Council for International Organizations of Medical Sciences (CIOMS) - 2016. International Ethical Guidelines for Health-Related Research Involving Humans. Available from: https://cioms.ch/wp-content/uploads/2017/01/WEB-CIOMS-EthicalGuidelines.pdf (Last accessed on 28th April 2020).
15. Nuffield Council on Bioethics. (2020). Rapid Policy Briefing. Ethical considerations in responding to the COVID-19 pandemic. 17th March 2020. Available from: https://www.nuffieldbioethics.org/assets/pdfs/Ethical-considerations-in-responding-to-the-COVID-19-pandemic.pdf (Last accessed on 28th April 2020).
16. Nuffield Council on Bioethics. (2020). Research in Global Health Emergencies: Ethical Issues. https://www.nuffieldbioethics.org/publications/research-in-global-health-emergencies (Last accessed on 28th April 2020).
17. COVID-19 Clinical Research Coalition. Global coalition to accelerate COVID-19 clinical research in resource-limited settings. The Lancet. Vol 395 April 25, 2020. Pg1322-25. Available from: https://www.dndi.org/wp-content/uploads/2020/04/Coalition-Accelerate-COVID19-Clinical-Research-Resource-Limited-Settings-TheLancet-2020.pdf (Last accessed on 28th April 2020).
18. WHO Laboratory biosafety guidance related to coronavirus disease (COVID-19). Interim guidance 19 March 2020. https://apps.who.int/iris/bitstream/handle/10665/331138/WHO-WPE-GIH-2020.1-eng.pdf?sequence=1&isAllowed=y (Last accessed on 28th April 2020).
19. Guidance for sample collection, packaging and transportation. Ministry of Health and Family Welfare. https://www.mohfw.gov.in/pdf/5Sample%20collection_packaging%20%202019-nCoV.pdf (Last accessed on 28th April 2020).
20. Novel Coronavirus Disease 2019 (COVID-19): Guidelines on rational use of Personal Protective Equipment. Ministry of Health and Family Welfare. https://www.mohfw.gov.in/pdf/GuidelinesonrationaluseofPersonalProtectiveEquipment.pdf (Last accessed on 28th April 2020).
21. Katharine Wright. COVID-19 and the ethical imperative of preparedness. Nuffield Council of Bioethics https://www.nuffieldbioethics.org/blog/covid-19-and-the-ethical-imperative-of-preparedness. (Last accessed on 28th April 2020)
22. Guidelines for Good Clinical Laboratory Practices (GCLP). Indian Council of Medical Research. 2008. https://www.icmr.gov.in/sites/default/files/guidelines/GCLP.pdf. (Last accessed on 28th April 2020)
23. Madhukar Pai. 'Covidisation' of academic research: opportunities and risks. Available from: https://naturemicrobiologycommunity.nature.com/users/20892-madhukar-pai/posts/65638-covidisation-of-academic-research-opportunities-and-risks. (Last accessed on 28th April 2020)
24. Regulations and Guidelines on Biosafety of Recombinant DNA Research and Biocontainment, 2017, Department of Biotechnology, Min. of Science and technology, Govt. of India. Available from: http://dbtindia.gov.in/sites/default/files/uploadfiles/Regulations_%26_Guidelines_for_Reocminant_DNA_Research_and_Biocontainment%2C2017.pdf (Last accessed on 28th April 2020).
25. Psychosocial Issues among Migrants during COVID-19, Ministry of Health and Family Welfare, Govt. of India. https://www.mohfw.gov.in/pdf/RevisedPsychosocialissuesofmigrantsCOVID19.pdf (Last accessed on 4th May 2020)
26. Review Committee on Genetic Manipulation, Department of Biotechnology, Govt. of India. https://ibkp.dbtindia.gov.in/Content/Commitee (Last accessed on 4th May 2020)

Annexure I: SOP Template for Ethics Review of Biomedical and Health Research during COVID-19 Pandemic

Institute Logo	**SOP for Review of Biomedical and Health Research during COVID-19 Pandemic**	**SOP No: __/ V01**<br>**Effective Date:** *dd/mm/yyyy*

1. **Purpose:**
   The purpose of this Standard Operating Procedure (SOP) is to describe how the EC will function and conduct ethics review in an emergency situation with restrictions as imposed by social distancing requirements during the COVID-19 outbreak.

2. **Procedures & Responsibilities:**

SN	Procedure	Responsibility
	**Submission and initial review**	
a.	Submit research proposal (electronically)	Researchers
b.	Receive, record, verify completeness and allot reference no.	Secretariat/ Member Secretary
c.	Categorize depending on risk (Exempt/ Expedited, Full committee), identify need for review by experts/ independent consultants/ patient /others, designate reviewers	Member Secretary in consultation with Chairperson
d.	Perform Initial review of documents as described in *Table 4.3 of ICMR National Ethical Guidelines*, fill study evaluation form	Primary/ secondary Reviewers
e.	Schedule virtual Meeting, Prepare Agenda, invite members (*Independent Consultants/Subject Experts/ PI/ Member secretary of local EC/ in consultation with Chairperson*).	Secretariat / Member Secretary
	**Virtual EC meeting**	
f.	Open the meeting, determine quorum (*Section 4.8.4 of ICMR National Ethical Guidelines), COI declaration, Summaries* Agenda	Chairperson
g.	Brief presentation and/or address queries on the research proposal and leave meeting prior to decision	Researchers/ subject experts (optional)
h.	Present observations on item reviewed	Primary/ secondary Reviewers
i.	Discuss further on the item and reach consensus	EC members
j.	Record Decision and rejoin member who had declared COI before moving on to subsequent item on agenda	Secretariat / Member Secretary
k.	Record minutes of meeting, ratify approved decisions of exemption/expedited review before closing meeting	Member Secretary/ Chairperson
	**Post meeting activities**	
l.	Communication of decision and maintaining records.	Secretariat/ Member Secretary
m.	Follow up/monitoring/ analysis of SAE/ handling of issues related to non-compliance, violation, complaints etc.	Member Secretary in consultation with Chairperson

3. **Detailed Instructions:**
   - The Research Proposal should be submitted electronically in ICMR Common Forms for Ethics Review (http://ethics.ncdirindia.org/Common_forms_for_Ethics_Committee.aspx) with supporting documents (Informed Consent, Brief CV of PI/ Co PIs, Questionnaire/ Case report form, Approval/ Comments of scientific committee, CTRI/ CDSCO/ HMSC/ MTA/ MoU/ insurance coverage) as applicable.
   - Once received, the secretariat will verify protocol for completeness (if not ask PI) and number.
   - Member Secretary to categorise research into full review, expedited review or exemption from review.
   - Member Secretary (in consultation with Chairperson) will identify need for review by subject experts, independent consultants, special invitees, patient representatives, others for prior review or to present views during the meeting.
   - The project for full review will be included in agenda of virtual full-committee meeting to be scheduled at the earliest (48 hrs) by the Member Secretary in consultation with the Chairperson.
   - The members will be briefed about the technological requirements and virtual platform used for the conduct of the meeting.
   - Quorum requirements for review will be applicable as per Section 4.8.4 ICMR National Ethical Guidelines, 2017.
   - Review procedures as per ICMR National Ethical Guidelines will also hold good for the virtual web ethics meeting.

4. **Annexures:** *if any*
5. **References:** ICMR National Ethical Guidelines for Biomedical and Health Research involving Human Participants

Prepared by	Reviewed by	Page **11** of **17**	Approved by	Accepted by
Signature with date	Signature with date		Signature with date	Signature with date

Impacting NCD Public Health Actions and Policies
Collaborate Innovate Inspire

ICMR Bioethics Unit

National Centre for Disease Informatics and Research
(Indian Council of Medical Research)
Kannamangala Post, Bangalore - 562 110, India.
http://ethics.ncdirindia.org
Email: icmr.bioethics@gmail.com
